# Raman Fingerprints of Rice Nutritional Quality: A Comparison between Japanese Koshihikari and Internationally Renowned Cultivars

**DOI:** 10.3390/foods10122936

**Published:** 2021-11-29

**Authors:** Giuseppe Pezzotti, Wenliang Zhu, Yuuki Hashimoto, Elia Marin, Takehiro Masumura, Yo-Ichiro Sato, Tetsuya Nakazaki

**Affiliations:** 1 Ceramic Physics Laboratory, Kyoto Institute of Technology, Sakyo-ku, Matsugasaki, Kyoto 606-8585, Japan; wlzhu@kit.ac.jp (W.Z.); tennis-0319-@outlook.com (Y.H.); elia-marin@kit.ac.jp (E.M.); 2 Department of Orthopedic Surgery, Tokyo Medical University, 6-7-1 Nishi-Shinjuku, Shinjuku-ku, Tokyo 160-0023, Japan; 3 The Center for Advanced Medical Engineering and Informatics, Osaka University, 2-2 Yamadaoka, Suita 565-0854, Japan; 4 Department of Immunology, Graduate School of Medical Science, Kyoto Prefectural University of Medicine, Kamigyo-ku, 465 Kajii-cho, Kyoto 602-8566, Japan; 5 Department of Dental Medicine, Graduate School of Medical Science, Kyoto Prefectural University of Medicine, Kamigyo-ku, Kyoto 602-8566, Japan; 6 Laboratory of Genetic Engineering, Kyoto Prefectural University, 1-5 Shimogamohangi-cho, Sakyo-ku, Kyoto 606-8522, Japan; masumura@kpu.ac.jp; 7 Research Center for Japanese Food Culture, Kyoto Prefectural University, 1-5 Shimogamohangi-cho, Sakyo-ku, Kyoto 606-8522, Japan; yisato@mtg.biglobe.ne.jp; 8 Experimental Farm, Graduate School of Agriculture, Kyoto University, Kizugawa 619-0218, Japan; nakazaki.tetsuya.4m@kyoto-u.ac.jp

**Keywords:** Raman spectroscopy, Koshihikari, internationally renowned rice cultivars, nutritional value, molecular fingerprints

## Abstract

Raman spectroscopy was applied to characterize at the molecular scale the nutritional quality of the Japanese Koshihikari rice cultivar in comparison with other renowned rice cultivars including Carnaroli from Italy, Calrose from the USA, Jasmine rice from Thailand, and Basmati from both India and Pakistan. For comparison, two glutinous (mochigome) cultivars were also investigated. Calibrated and validated Raman analytical algorithms allowed quantitative determinations of: (i) amylopectin and amylose concentrations, (ii) fractions of aromatic amino acids, and (iii) protein content and secondary structure. The Raman assessments non-destructively linked the molecular composition of grains to key nutritional parameters and revealed a complex intertwine of chemical properties. The Koshihikari cultivar was rich in proteins (but with low statistical relevance as compared to other investigated cultivars) and aromatic amino acids. However, it also induced a clearly higher glycemic impact as compared to long-grain cultivars from Asian countries. Complementary to genomics and wet-chemistry analyses, Raman spectroscopy makes non-destructively available factual and data-driven information on rice nutritional characteristics, thus providing customers, dietitian nutritionists, and producers with a solid science-consolidated platform.

## 1. Introduction

Unlike the notion of “rice quality”, which is heterogeneous and context-specific [1], the nutritional benefits of rice can be defined in the objectiveness and realm of molecular-scale food chemistry. Food scientists have not yet reached unanimity on selecting a specific nutritional framework, but several parameters related to molecular composition are known to clearly define the nutritional value of rice cultivars [2]. Parameters so far proposed include concentrations of different polysaccharide structures (i.e., amylopectin-to-amylose ratio directly related to glycemic index), protein-to-carbohydrate ratio, and fraction of antioxidant aromatic amino acids (i.e., phenylalanine and tryptophan). The rice nutritional traits can partly be predicted through genetics, as multiple sets of genes interact with each other and induce additive, dominant, epistatic, or pleiotropic effects on the biochemical pathways that ultimately lead to the observed rice characteristics [3]. However, the multiple interacting genes that control rice nutritional characteristics are invariably expressed through local and contingent environmental conditions [4]. Therefore, evaluations of nutritional values for specific cultivars necessarily require that genetic information be crossed with compositional phenotypes related to seasonal growing conditions and aging during storage.

Given the strong environmental effects on rice nutritional value, the definition of “premium quality” for rice in terms of factual nutritional arguments requires a molecular chemistry approach parallel to genomics. Rice nutritional quality arises from a bundle of intrinsic and extrinsic attributes and can only be quantitatively defined through analyses at the molecular scale. Moreover, being rice traded internationally, standardized parameters should be set for comparing different cultivars (eventually with the same genetic traits but) harvested in different environments and/or stored for different times. Food technologists have not yet reached a consensus on quality classes and measurement methods, so that quantitative indicators and routine metrics for addressing raw-grain quality at the molecular scale are yet conspicuously missing. In this context, Raman spectroscopy offers a unique chance for fast, quantitative, and non-destructive evaluations of rice nutritional value in real time and at low cost [5,6]. Being a “fingerprint” of molecular composition, the Raman spectrum embodies a large amount of structural information. However, Raman assessments require specific calibrations with basic compounds to become fully quantitative. Once such calibrations are established, a full set of nutritional parameters could become available at once and non-destructively.

This article aims at providing comparative Raman spectroscopic analyses of the nutritional characteristics of internationally recognized rice cultivars. It builds upon and further refines previously presented Raman analytical algorithms [7,8] for the determination of amylopectin/amylose, aromatic amino acids, and grain protein concentrations, which directly represent glycemic, antioxidative/taste-sensory, and nutritional characteristics of rice cultivars, respectively. As representative for the Japanese rice cultivars, we selected the popular short-grain Koshihikari cultivar [9]. With its long tradition, the Koshihikari rice has gained high recognition and met the eating preferences of many Japanese and international consumers. The main reasons for the widespread preference for Koshihikari cultivar among Japanese consumers reside in its sticky/chewy texture and sweet/nutty taste. On the other hand, the rice cultivars selected for comparison included renowned cultivars such as short-grain Carnaroli from Italy, short-grain Carlrose from the USA, long-grain Jasmine rice and mochigome from Thailand, and long-grain Basmati from both India and Pakistan. These rice cultivars were selected because of their different texture and taste characteristics as compared to the Koshihikari cultivar [10].

The short-grain Koshihikari cultivar was developed in 1956 and yet still enjoys the top market share percentage in Japan, despite the increasingly fast and wide diversifications of the eating habits for cooked rice among young Japanese consumers. When cultivated in different Japanese prefectures, it turns into compositional phenotypes that differ depending on the climate characteristics of their regions of provenience, but yet preserve some common key characteristics [11]. The choice of the Koshihikari Kyoto cultivar (simply referred to as Koshihikari, henceforth) for this study was based both on the popularity of this cultivar in Japan and on its being used as a benchmark in rating the characteristics of newly developed Japanese rice cultivars. The Carnaroli cultivar was developed in Italy in 1945. This cultivar is a medium size kernel rice renowned for its creamy texture after cooking. This peculiarity arises from the characteristic of being high in amylopectin content, whose dissolution thickens the surrounding cooking liquid, a special feature required for the notorious *risotto* dishes. However, a standardized evaluation of gelatinization time of rice kernels upon cooking, which was carried out by comparing ten different Italian rice varieties [12], showed that the Carnaroli cultivar experienced the third longer gelatinization (and cooking) time among the investigated cultivars. Grown in California, the Calrose cultivar has a peculiar characteristic: once cooked, it tends to become soft and slightly sticky, which makes it known as “general purpose” rice. Developed in 1948 [13,14], it has promptly become a prominent rice variety in California where it represents ~80% of the rice crop production. The development of Basmati cultivars is believed to trace back to several centuries. Its typical aroma, usually referred to as the *pandan*-like flavor, is due to the aroma compound 2-acetyl-1-pyrroline, which in Basmati rice is present at levels 12 times higher than any non-Basmati rice species [15]. Another general peculiarity of all Basmati cultivars is the low glycemic impact they induce as compared to other rice species. Jasmine rice becomes moist and soft in texture upon cooking, while retaining a slightly sweet flavor. It is also relatively stickier than other long-grain rice species. This characteristic is in agreement with the finding that its glycemic impact was reported to be higher than that of Basmati [16].

This study was motivated by the observation that the Koshihikari, similar to other Japanese rice cultivars, has kept through the years a unit price more than threefold those of other internationally renowned cultivars [11]. Measured and defined in the realm of molecular food science and parallel to genetics, we apply here quantitative Raman spectroscopy to assess whether such a remarkable difference in price is accompanied by equally superior nutritional characteristics. Quantitative Raman algorithms reveal how the Japanese Koshihikari cultivar differs from selected popular rice cultivars from the US, Europe, Central and South Asia.

## 2. Materials and Methods

### 2.1. Rice Samples

**Figure 1:**
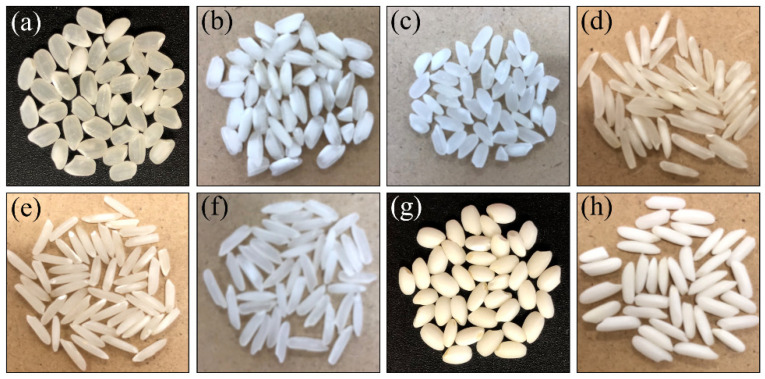
Photographs of rice kernels for Koshihikari and other international rice cultivars investigated in this study: (**a**) short-grain Koshihikari (Japan), (**b**) short-grain Carnaroli (Italy), (**c**) short-grain Calrose (USA), (**d**) long-grain Basmati (India), (**e**) long-grain Basmati (Pakistan), (**f**) long-grain Jasmine (Thailand), (**g**) short-grain glutinous Mochigome (Japan), and (**h**) long-grain glutinous Mochigome (Thailand).

Figure 1a–h shows photographs of rice kernels from the Koshihikari and other rice cultivars investigated in this study. The studied Koshihikari cultivar (Figure 1a), which was harvested in Kyoto Prefecture, has gained popularity for its peculiar texture and flavor, both retained even after long-term storage. The premium rice cultivar Carnaroli (Figure 1b) was grown in the Pavia province of northern Italy. The Calrose cultivar from California (USA, Figure 1c) is a japonica rice characterized by medium size kernel morphology. The Basmati cultivar is a long-grained aromatic rice variety geographically exclusive to specific districts of India (Figure 1d) and Pakistan (Figure 1e) [14]. The investigated Jasmine rice (Figure 1f) from Thailand is a long-grained aromatic rice cultivar with pandan-like flavor similar to the Basmati rice. For comparison, two glutinous rice (mochigome) cultivars were also investigated, one from Japan (Figure 1g) and one from Thailand (Figure 1h). The Japanese one is referred to as the “Shinhabutaemochi” and is harvested in Kyoto Prefecture. According to X-ray diffraction data published by Zhang et al. [17], a main characteristic of this cultivar is the relatively high content of monoclinic phase in its amylose structure. The mochigome cultivar from Thailand (khao niao type from central Thailand) was morphologically very different from the Japanese one (long-grained vs. short-grained). This latter kind of glutinous rice is very popular because used in a number of dishes in the traditional Thai cuisine, while being the staple food for people in the north and northeast of Thailand. All rice cultivars investigated in this study were harvested in the year 2019.

Note that, as the investigated international rice samples were purchased from an import store in Japan, the authors have no direct guarantee on the authenticity of the tested cultivars. However, while the actual origin of the investigated samples could in principle be a concern, the Japanese regulations on the origin of imported food, usually referred to as Rules of Origin (ROO), are very strict. ROO is regulated by the Act on Record of Transaction Information and Dissemination of Origin Information of Rice, which identify not only the origin but also the entire distribution route based on preserved records. These strict procedures ensure proper labeling, authenticity, and safety of imported rice. The so-called Rice Traceability System in Japan mandatorily requires detailed and proved information on places of production. For the above body of reasons, the authors believe that the tested samples are authentic.

### 2.2. Raman Spectroscopic Assessments

Raman spectra were non-destructively collected on grain kernels of different rice cultivars without grinding or other manipulations. We used a high-resolution Raman spectroscope, which was equipped with a triple monochromator (T-64000, Horiba/Jobin-Yvon, Kyoto, Japan) and operated in microscopic mode with a 20× optical lens. The achieved spectral resolution was 0.1 cm^−1^. The blue line of a 488 nm Ar-ion laser (Stabilite 2017, Spectra Physics, Mountain View, CA, USA) was used as an excitation source and applied with a power of 10 mW. The Raman light was diffracted into a monochromator connected with an air-cooled 1024 × 256 pixels charge-coupled device (CCD) detector (CCD-3500V, Horiba Ltd., Kyoto, Japan). The acquisition time for one spectrum was 30 s. Average of 30 spectra collected on 30 different grains for each sample were analyzed. Baseline correction was performed with subtracting a line segment. A mixed Gauss-Lorentz function was selected for band deconvolution because the laser probe for Raman measurements incorporates an in-plane Gaussian distribution of intensity [18]:

(1) $$I\left(\omega \right)=A\;\left(g\times \frac{1}{\sqrt{2\pi }w}\mathrm{Exp}\left[-\frac{{\left(\omega -\omega_{p}\right)}^{2}}{2w^{2}}\right]+\left(1-g\right)\times \frac{1}{{\left(\omega -\omega_{p}\right)}^{2}+w^{2}}\right)$$

where *ω_p_*, *w* and *A* are the peak position, width and area, respectively, and *g* is a parameter showing the fraction of Gauss function (*g* = 0.5 in this study).

Both baseline correction and band deconvolution procedures were performed using commercially available software (LabSpec 4.02, Horiba/Jobin-Yvon, Kyoto, Japan). A reiterative fitting algorithm refined the sub-band choices for a pre-determined number of times (5 × 10^7^ times) in order to minimize the difference between the sum of the selected sub-bands and the experimental curve. The above number of reiterations was a fixed number in the program and set the upper value of reiterations performed by the fitting algorithm for solution acceptance. The acceptance criterion for curve matching was set to a confidence better than 95%. The penetration depth of the laser probe into rice kernel samples was assessed by means of a probe-response function procedure using the defocussing method [19]. The Raman probe penetration depth did not significantly vary among the tested samples and was typically in the order of 0.2–0.3 mm for glutinous, unpolished, and polished rice kernels. Analyses on isolated grains, which were completely non-destructive, were conducted by focusing the laser spot on the outer skin of the grains. However, given the above-shown penetration depth of the incoming laser, the Raman signal also comprised internal contributions from the first few hundreds of microns toward the endosperm. This procedure was adopted with the purpose of developing a comparative Raman protocol for non-destructive assessments of rice nutritional traits.

### 2.3. Statistical Analyses

The experimental data were analyzed with respect to their statistical meaning by computing their mean value ± one standard deviation. Student’s *t*-test was applied with *p* values < 0.05 being considered statistically significant and labeled with one asterisk.

## 3. Results

### 3.1. Raman Spectra of Koshihikari and Other Renowned Cultivars

**Figure 2:**
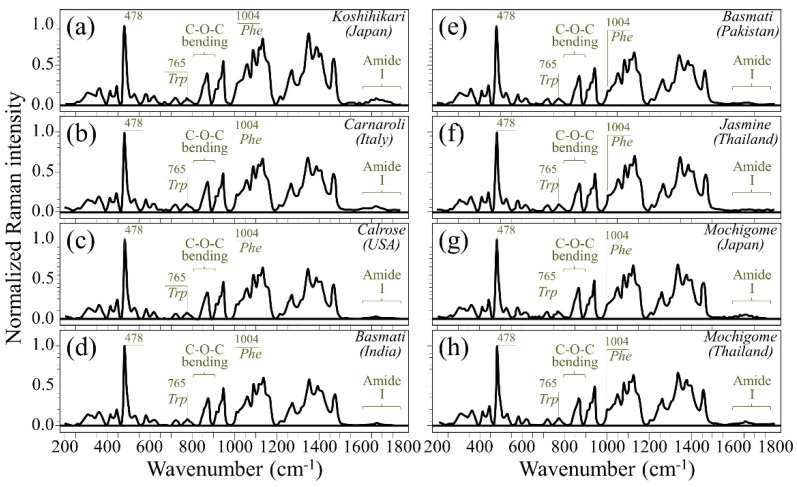
Low-resolution Raman spectra collected on as-received kernels of eight different rice cultivars (cf. labels in inset) in the spectral region 200–1800 cm^−1^; the spectra are averaged over 30 spectra per each type of cultivar and normalized to the glucose ring stretching band at ~478 cm^−1^ (cf. label in inset). The abbreviations *Phe* and *Trp* refer to phenylalanine and tryptophan, respectively, while the wavenumbers in inset are given in cm^−1^ units. Insets: (**a**) Koshihikari (Japan), (**b**) Carnaroli (Italy), (**c**) Calrose (USA), (**d**) Basmati (India), (**e**) Basmati (Pakistan), (**f**) Jasmine (Thailand), (**g**) Mochigome (Japan) and (**h**) Mochigome (Thailand).

Figure 2a–h shows low-resolution Raman spectra collected on as-received kernels of the eight different rice cultivars shown in Figure 1 (cf. labels in inset). All spectra, which were collected in the wavenumber region 200–1800 cm^−1^ and represent averages of 30 spectra per each type of cultivar, were normalized to the glucose ring stretching band at ~478 cm^−1^. This signal was selected for normalization because common to all carbohydrate polymers while preserving a constant morphology and showing negligible shifts in frequencies for different rice samples. Raman signals from polysaccharides, which appear below 500 cm^−1^, at 950–1200 cm^−1^, and at 1200–1500 cm^−1^, represent skeletal breathing modes, coupled C–C and C–O symmetric stretching modes, and CH deformation modes, respectively [20]. On the other hand, the spectral region between 800 and 900 cm^−1^ is representative of C–O–C bending modes (cf. labels in Figure 2), which differ between amylose and amylopectin structures. As shown later, deconvolution of this spectral zone into sub-bands enables the computation of the relative fractions of different polysaccharides. Note that a difference in the number of branches in the amylopectin structure slightly alters the Raman band positions. However, such small variations in wavenumber have no tangible effect on the computed fraction of amylopectin.

A sharp and relatively strong Raman line at ~1003 cm^−1^ is characteristic of benzene ring breathing in aromatic amino acid phenylalanine (cf. Figure 2). This signal, which is seen as a shoulder in the spectra of all the investigated cultivars, can be used to quantitatively characterize the weight fraction of this aromatic amino acid in rice kernels, provided that a quantitative equation is built up through preliminary calibrations with known fractions. With a similar procedure, also the fraction of tryptophan can be retrieved from the Raman spectrum by analyzing the Raman signal at ~768 cm^−1^. This signal arises from breathing vibrations of the indole ring and represents a peculiar feature of the aromatic amino acid tryptophan (cf. Figure 2). Quantifications of aromatic amino acid weight fractions will be discussed in detail in a later section.

Another important spectral feature in the context of rice nutritional value is the Amide I signal (cf. Figure 2). This signal represents a composite vibrational mode combining C=O stretching, C–N stretching, and N–H bending modes, all occurring within the so-called amide plane of the protein structure. The Amide I spectral zone covers the interval 1620–1700 cm^−1^ and appears to be quite weak as compared to the other spectral features of rice (cf. Figure 2). However, when collected with a high resolution spectroscope (as shown later), it can be deconvoluted into four sub-bands characteristic of the secondary structure of proteins, as follows: β-sheet at 1636–1640 cm^−1^, α-helix at 1658–1662 cm^−1^, random coil at 1674–1693 cm^−1^, and β-turn at 1695–1697 cm^−1^ [21,22,23]. After performing accurate calibrations, the Raman intensity ratio between the maximum intensity in the Amide I zone at ~1670 cm^−1^ (characteristic of proteins) and the glucose ring signal at 478 cm^−1^ (characteristic of all polysaccharides) can be used as a quantification of protein weight fraction. Moreover, the Raman spectral profile of the Amide I signal can be used for quantifying the secondary structure of proteins by computing the relative (areal) intensity of the above sub-bands [23]. Detailed analyses of protein-to-carbohydrate ratio and protein structure in rice kernels based on the above approaches are presented in the next Section 3.2.

### 3.2. Quantitative Evaluation of Nutritional Quality of Rice Cultivars

Three parameters were selected for evaluating and comparing the nutritional value of rice cultivars, as follows: (i) the amylose (vs. amylopectin) fraction, (ii) the weight fractions of phenylalanine and tryptophan aromatic amino acids, and (iii) the protein weight ratio. In addition, also the secondary structure of proteins was analyzed as an additional parameter because of its impact on protein solubility and digestibility, as further discussed in Section 4.

**Figure 3:**
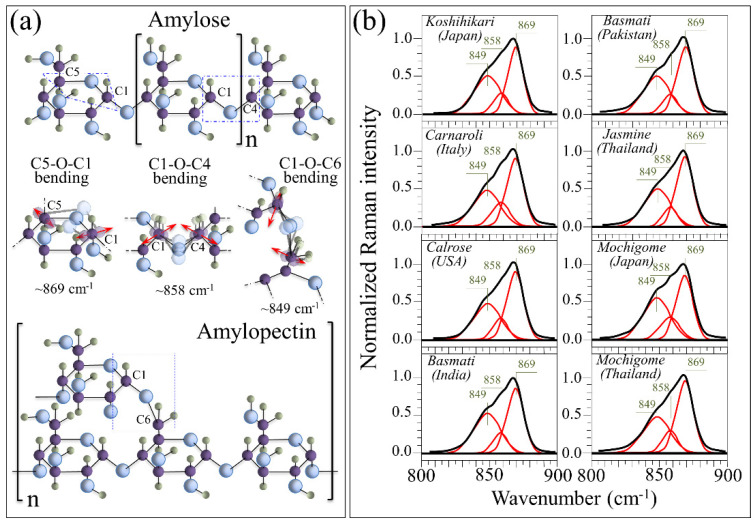
(**a**) Structures and the C–O–C bending modes of amylose and amylopectin; C–O–C bending modes include intra-ring C5–O–C1, inter-ring C1–O–C4 and C1–O–C6 bending vibrations found at ~869, ~858, and 849 cm^−1^, respectively. In (**b**), high-resolution Raman spectra in the 800–900 cm^−1^ C–O–C bending region as collected on eight different rice cultivars from Japan and other countries (cf. labels in inset); deconvolutions into three sub-bands centered at 869, 858, and 849 cm^−1^ fit the experimental signals with a confidence >95%.

Amylose and amylopectin share vibrational modes of their common polymeric glucose-ring structures, but their different molecular assemblies lead to spectral variations that can be used to quantify their respective fractions in rice kernels. Figure 3a shows the different structures and the C–O–C bending modes in amylose and amylopectin. In two previous studies [7,8], we proposed and validated a quantitative algorithm for computing the fractions of amylose and amylopectin based on spectral differences in the wavenumber interval 830–895 cm^−1^, which represents the C–O–C bending modes. As seen in Figure 3a, the intra-ring C5–O–C1 and the inter-ring C1–O–C4 bending vibrations, which can be found in both types of polysaccharide structures, give rise to two sub-bands centered at ~869 and ~858 cm^−1^, respectively. On the other hand, the C1–O–C6 bending vibration, which scatters at lower wavenumbers ~849 cm^−1^, can only be found in the amylopectin structure owing to its peculiar branched structure. In Figure 3b, high-resolution Raman spectra in the 800–900 cm^−1^ spectral region are shown as collected on eight different rice cultivars from Japan and other countries (cf. labels in inset). The Raman spectra in the C–O–C bending zone were deconvoluted into three sub-bands (at 869, 858, and 849 cm^−1^) to fit the experimental signals according to the reiterative algorithm described in Section 2.2. Note that in the deconvolution process, to avoid physically meaningless fitting due to the non-resolvable nature of this broad band, both band position and width of the three sub-bands were “constrained”; i.e., the band position and width were treated as unknown parameters for spectral fitting, but a boundary condition was set that the same sub-band should have same band position and width for all samples.

**Figure 4:**
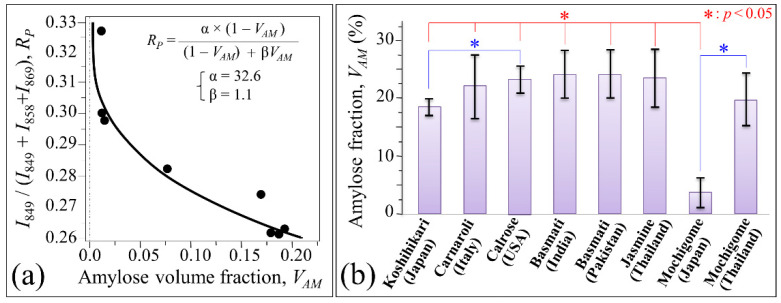
(**a**) Calibration curve relating the spectral intensity ratio of C–O–C bending sub-bands, *R_P_* = *I*_849_/(*I*_849_ + *I*_858_ + *I*_869_), to the amylose volume fraction, *V_AM_*; least square fitting equation and related constant in inset. In (**b**), average values in percents of amylose fractions measured in Japanese and international rice cultivars (standard deviations computed over 30 measurements at different locations per each type of rice) and statistical validation according to Student’s *t*-test (cf. label in inset).

Signals in the C–O–C bending zone showed clear variations both in intensity and morphology for different rice cultivars as a consequence of the different relative intensities of their sub-band components. Upon calibrating the relative intensity of the C1–O–C6 Raman signal with respect to the C5–O–C1 and C1–O–C4 ones, it is possible to set an algorithm that locates the percent volume fractions of amylose, *V_AM_*, and amylopectin, *V_AP_*, from Raman spectroscopic assessments of rice kernels. Figure 4a shows a calibration curve, which was constructed to relate the spectral intensity ratio of the above-mentioned three sub-bands, *R_P_* = *I*_849_/(*I*_849_ + *I*_858_ + *I*_869_), to amylose volume fraction, *V_AM_* [7,8]. In Ref. [7], the Raman calibration of amylose content was validated by iodine colorimetry experiments in duplicate on 60-mesh milled rice flour by iodine colorimetry, according to ISO standard [24]. In this latter validation procedure, the iodine absorption spectrum was recorded and analyzed in the interval 200–900 nm using distilled water was as a reference. In the experimental calibration, the sub-band intensities, *I*, were computed from the areas subtended by the three respective C–O–C bending components (cf. Figure 3b). The fractions of amylose, *V_AM_*, and amylopectin, *V_AP_*, can be computed from the knowledge of the *R_P_* ratio according to the following Equations (2) and (3):

V_AM_ = (α − R_P_)/[α + R_P_(β − 1)] (2)

V_AP_ = 1 − V_AM_ (3)

where α and β are two numerical parameters obtained from fitting the experimental calibration curve (cf. labels in inset to Figure 4a)

In Figure 4b, average values of (percent) amylose fractions are plotted for the Koshihikari and other rice cultivars as computed from their respective (deconvoluted) Raman spectra in Figure 3b. Standard deviations are also shown as obtained over 30 measurements at different locations per each type of rice. The results of Student’s *t*-test (also shown in Figure 4b) revealed statistically significant differences between Japanese and other international cultivars. As generally expected for glutinous rice cultivars, the Japanese mochigome (Shinhabutaemochi) was almost completely composed of amylopectin (i.e., it only contained few percents of amylose). However, the Thai mochigome (*khao niao*), despite its glutinous nature, did not statistically differ from the other investigated non-glutinous cultivars regarding its amylose content (~19%). The Japanese Koshihikari cultivar was the cultivar with the lowest average volume fraction (~18%) of amylose (i.e., ~82% content of amylopectin) among the investigated non-glutinous cultivars. However, this difference was statistically validated only with respect to the USA Calrose (~22% amylose, ~78% amylopectin), which also showed a relatively low standard deviation among the investigated samples. The highest average values of amylose fraction (~24%) were recorded in the three types of long-grain rice cultivars, namely, two Basmati types from India and Pakistan, and the Thai Jasmine cultivars. On the other hand, the Italian Carnaroli, despite having an average amylose fraction (~22%) higher than the Japanese Koshihikari, showed the highest standard deviation among the measured cultivars with minimum *V_AM_* values as low as ~16%. In other words, the Carnaroli cultivar was the most inhomogeneous cultivar investigated with respect to carbohydrate composition.

While the presented Raman measurements of amylose/amylopectin fractions well matches both qualitative descriptions and consumers perception of the glutinous, sticky, and long-grain aromatic types of rice found in general rice classifications [25], the presented spectroscopic characterizations also give rigorous quantifications of composition and homogeneity of the rice carbohydrate structure.

**Figure 5:**
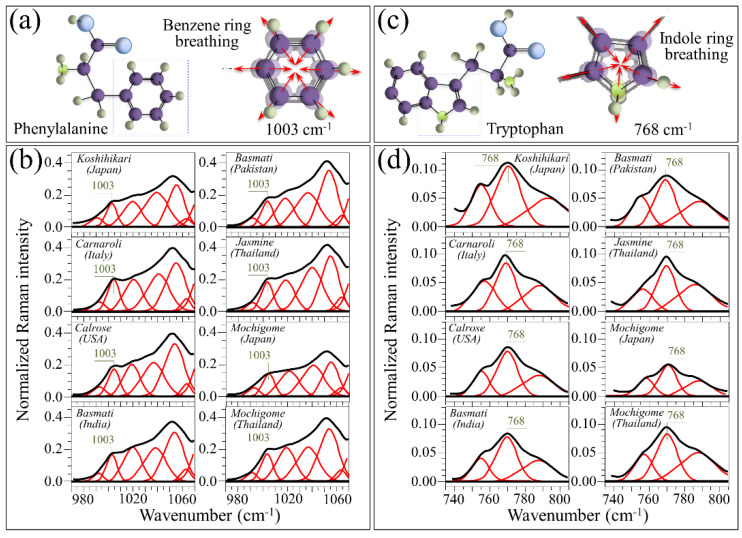
(**a**) Draft of the molecular structure of phenylalanine and breathing vibrations of its benzene ring (Raman line at ~1003 cm^−1^), and (**b**) high-resolution (deconvoluted) Raman spectra of different rice cultivars (cf. labels in inset) in the spectral region 970–1070 cm^−1^; (**c**) draft of the molecular structure of the aromatic amino acid tryptophan and its indole ring breathing vibration at ~768 cm^−1^, and (**d**) high resolution (deconvoluted) Raman spectra of different rice cultivars (cf. labels in inset) in the region 735–805 cm^−1^.

**Figure 6:**
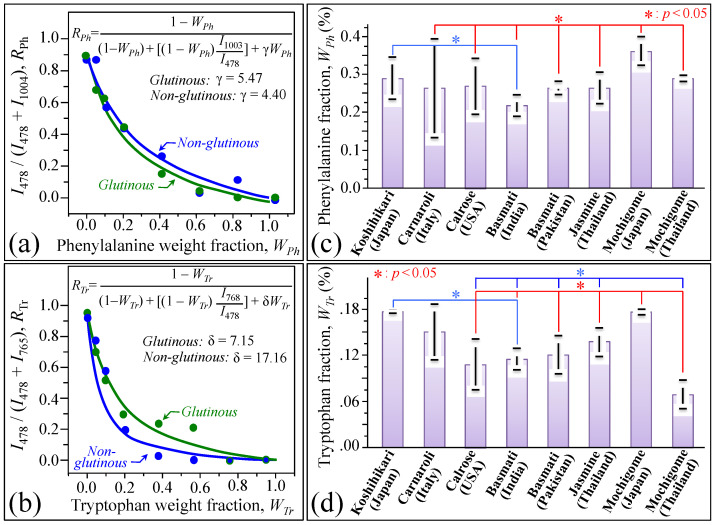
Raman calibration plots of ring vibrational signals (normalized to glucose ring stretching band at ~478 cm^−1^) vs. weight fractions of (**a**) phenylalanine (Raman sub-band ratio *R_Ph_* = *I*_1003_/*I*_478_ vs. *W_Ph_*) and (**b**) tryptophan (Raman sub-band ratio *R_Tr_* = *I*_768_/*I*_478_ vs. *W_Tr_*); fitting equations and related constant are given in inset. In (**c**,**d**), percent weight fractions of phenylalanine and tryptophan, respectively; standard deviations are computed over 30 measurements at different locations per each type of rice and statistical validation according to Student’s *t*-test according to labels in inset.

The benzene ring of phenylalanine presents a distinct Raman line at ~1003 cm^−1^ characteristic of its breathing mode (Figure 5a) [22]. Although this band is seen as a shoulder in the Raman spectrum of rice kernels, it can clearly be deconvoluted by fixing its full width at half maximum (as obtained from the Raman spectrum of pure phenylalanine) and can be used to quantitatively characterize the fractional amount of this aromatic amino acid. In Figure 5b, high-resolution (deconvoluted) Raman spectra are shown for different rice cultivars (cf. labels) in the spectral region 970–1070 cm^−1^. In this spectral zone, the 1003 cm^−1^ band is partly overlapping other band components corresponding to C-O and C-C stretching in carbohydrates [20]. However, differences in its relative intensity can clearly be noticed among different cultivars. Figure 5c shows a draft of the molecular structure of the aromatic amino acid tryptophan and its peculiar indole ring breathing vibration at ~768 cm^−1^ [22]. According to a procedure similar to the Raman analysis of phenylalanine, highly resolved Raman spectra of different rice cultivars were deconvoluted in the region 735–805 cm^−1^ (Figure 5d). The deconvoluted spectra presented three partly overlapping sub-bands, the stronger signal of this triplet being the central component from indole ring vibration. The remaining two components at lower and higher wavenumbers relate to CH_2_ rocking and C–O–C stretching in amylose and amylopectin, respectively [20]. Quantifications of both phenylalanine and tryptophan fractions become possible provided that accurate calibrations are preliminarily conducted with known fractions of these aromatic amino acids in order to build up viable and quantitative Raman equations. The results of Raman calibrations of fractional contents are shown in Figure 6a,b for phenylalanine and tryptophan, respectively (cf. also fitting equations in insets). The plots link relative Raman intensity of ring vibrational signals with respect to the glucose ring stretching band at ~478 cm^−1^ (i.e., the areal intensity ratios *R_Ph_* = *I*_1003_/*I*_478_ and *R_Tr_* = *I*_768_/*I*_478_ for phenylalanine and tryptophan, respectively) to the respective weight fractions, *W_Ph_* and *W_Tr_*. Similar to the above-described case of polysaccharide calibration, sub-band intensities, *I*, were computed from the areas subtended by the respective band components. The weight fractions of phenylalanine and tryptophan as a function of spectral ratios *R_Ph_* and *R_Tr_* obeyed the following Equations (4) and (5), respectively:

W_Ph_ (%) = [R_Ph_/(γ + R_Ph_)] × 100 (4)

W_Tr_ (%) = [R_Tr_/(δ + R_Tr_)] × 100 (5)

where γ and δ are two numerical constants obtained from fitting the experimental calibration curves (cf. labels in inset to Figure 6a,b, respectively).

Based on the above quantitative calibrations, Figure 6c,d show average weight fractions (in percent) of phenylalanine and tryptophan, respectively, as measured from the deconvoluted Raman spectra of Japanese and foreign rice cultivars (cf. Figure 5b,d, respectively). Standard deviations are also shown as computed over 30 measurements at different locations per each type of rice. The plots in Figure 5b,d show that the Japanese mochigome and the Koshihikari Japanese cultivars presented the highest fractions of aromatic amino acids. This peculiar characteristic was statistically validated with respect to the other investigated rice cultivars except for the Italian Carnaroli. The Carnaroli cultivar showed statistically non-significant differences in aromatic amino acids with respect to both Japanese cultivars, but also a relatively high standard deviation in the case of phenylalanine content (cf. Figure 5b). Long-grain cultivars generally showed a relatively low fraction of aromatic amino acids, except for the Thai mochigome, which was relatively rich in phenylalanine but also the poorest in tryptophan. A similar trend was also observed for the Calrose cultivar. Despite their importance as building blocks for proteins and precursor molecules in secondary metabolism, phenylalanine and tryptophan cannot be synthesized in the human body. Their supply only depends on nutritional provision. Therefore, the presence of these essential amino acids in rice kernels should be considered as an important nutritional characteristic for rice cultivars.

**Figure 7:**
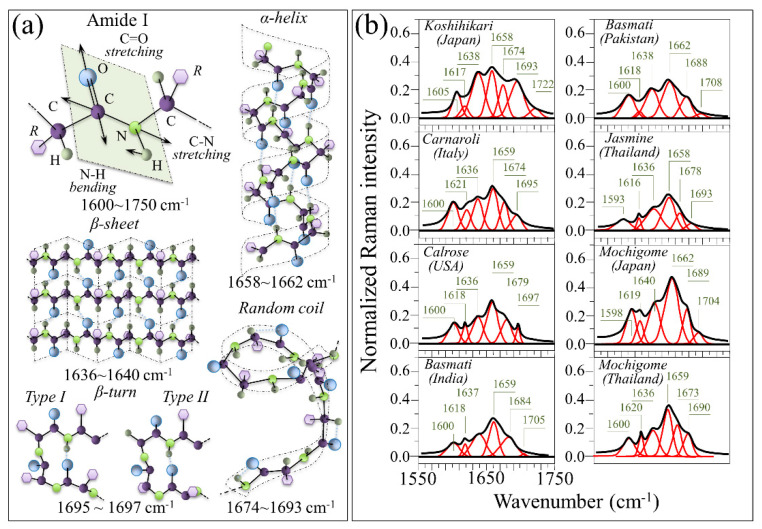
(**a**) The Amide I vibrational mode and schematic drafts of protein secondary structures including β-sheet, α-helix, random coil, and β-turn (related Raman frequencies in inset); (**b**) deconvoluted Raman spectra in the Amide I region (i.e., 1550–1750 cm^−1^) for the eight investigated rice cultivars (cf. labels in inset). The spectra are normalized to their respective glucose ring stretching signals at ~478 cm^−1^ and wavenumbers in inset are given in cm^−1^ units.

Figure 7a shows the Amide I vibrational mode and schematic drafts of protein secondary structures including β-sheet, α-helix, random coil, and β-turn. In inset, Raman frequencies are shown according to the specific secondary structure in which the Amide I vibrations originates. In Figure 7b, deconvoluted Raman spectra in the Amide I region (i.e., 1550–1750 cm^−1^) are shown for the eight investigated rice cultivars (cf. labels in inset). The shown spectra were normalized to their respective glucose ring stretching signals at ~478 cm^−1^. Significant morphological differences can be noticed among different cultivars. The 1550–1750 cm^−1^ spectral zone in Figure 7b could be deconvoluted into 6 sub-bands, except for the Japanese mochigome, which showed seven sub-bands due to the presence of an additional band at high frequencies. The Amide I signals, which were typically located in the spectral ranges 1636–1640, 1658–1662, 1674–1693, and 1695–1697 cm^−1^, were assigned to β-sheet, α-helix, random coil, and β-turn secondary structures, respectively [21,22,23,26]. Two partly overlapping sub-bands located at lower frequencies (i.e., 1593–1605 and 1616–1621 cm^−1^) belonged to phenylalanine and tryptophan/tyrosine side chains, respectively [22,27]. The weak band seen beyond 1700 cm^−1^ can be assigned to C=O stretching. Discussed in detail in a previous work [8], this band represents an alteration of the lysine residue induced by succinylation [28]. As a general feature for all investigated cultivars, the signal representing β-sheet was weaker than that representing α-helix, which was the most pronounced signal recorded in the Amide I spectral zone. However, the relative intensity of the α-helix signal greatly varied among different cultivars. Among non-glutinous rice cultivars, the strongest Amide I signal could be found in the Japanese Koshihikari cultivar, while the lowest one was recorded in the Thai Jasmine one. Note that the Japanese Koshihikari cultivar was also the cultivar with the lowest relative intensity of α-helix with respect to both β-sheet and random coil signals among all the investigated non-glutinous rice cultivars. The Japanese and Thai mochigome cultivars displayed comparatively strong α-helix signals, while only the latter showed a relatively strong β-turn contribution to the overall protein structure. These characteristics of the protein secondary structure will be discussed in the next section.

**Figure 8:**
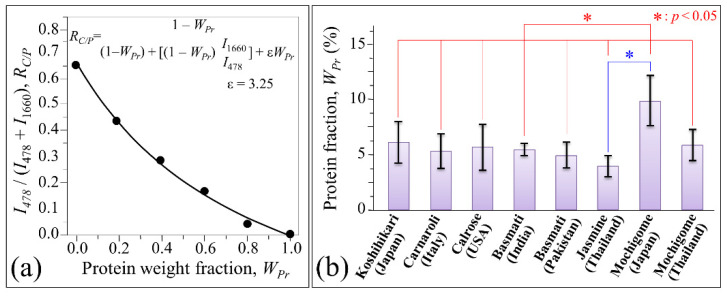
(**a**) Calibration plot of the Amide I Raman intensity ratio, *R_C/P_* = *I*_478_/(*I*_478_ + *I*_1660_), vs. protein weight fraction, *W_Pr_* (least-square fitting equation in inset); (**b**) protein weight fraction computed from Raman experiments for the eight investigated rice (cf. labels).

In order to obtain a quantification of the protein weight fraction, *W_Pr_*, contained in the studied rice cultivars, an accurate calibration was conducted with adding known protein fractions to pulverized rice kernels. This preliminary calibration procedure, whose results are shown in Figure 8a, allowed us to build up a viable algorithm to compute the *W_Pr_* parameter from Raman analyses. The plot links *W_Pr_* to the Raman intensity ratio between the glucose ring signal at ~478 cm^−1^ (representative of all carbohydrates) and the maximum intensity of the Amide I signal at 1658–1662 cm^−1^ (labeled as 1660 cm^−1^), *R_C/P_* = *I*_478_/(*I*_478_ + *I*_1660_). The (least-square) fitting equation is given in inset. The protein fractional contents computed for all investigated rice cultivars are shown in Figure 8b. The protein fractions contained in the rice kernels obeyed the following dependence on Raman intensity ratio, *R_Pr_* = *I*_1660_/*I*_478_:

W_Pr_ (%) = [R_Pr_/(ε + R_Ph_)] × 100 (6)

where *R_Pr_* = I_1660_/I_478_ and ε is a numerical constant obtained from fitting the experimental calibration curve (cf. label in inset to Figure 8a).

Figure 8b reveals important differences in protein content among the investigated cultivars. Standard deviations (based on computations over 30 measurements at different locations per each cultivar) served to locate the statistical validity of the obtained data. The Japanese Koshihikari and the Thai Jasmine cultivars contained the highest (5.7%) and the lowest (4.0%) (average) weight fractions of proteins. However, due to the high standard deviations recorded, no statistical relevance could be found between any of the investigated non-glutinous rice cultivars. On the other hand, the Japanese mochigome was by far the highest in protein content with a striking 8.8%. Significantly higher than the Thai mochigome (5.7%), it also scored statistically significant difference with respect to all other investigated cultivars.

## 4. Discussion

### 4.1. Glycemic Impact of Glutinous and Non-Glutinous Rice Cultivars

The digestion of starch through enzymatic reactions originates a rapid release of glucose rings into the bloodstream. Postprandial glycemic control thus plays a fundamental role in preventing diabetes and slowing its complications. Clinical studies of glycemic index (GI), which classifies the glucose-raising effect of carbohydrate containing foods with respect to pure glucose or white bread, Ref. [29] have shown that foods with similar carbohydrate contents might have quite different impacts on blood glucose levels [30,31]. In this context, a slowly digestible starch (or resistant starch) is the most suitable for the prevention and management of diabetes, because it reaches largely undigested the colon, thus enabling a better control of blood glucose levels [32]. Slow digestibility of rice starch is tightly correlated with its amylose content [33]. Accordingly, the development of rice cultivars with slow-digesting characteristics (i.e., rich in amylose) is of fundamental importance in our modern society.

**Figure 9:**
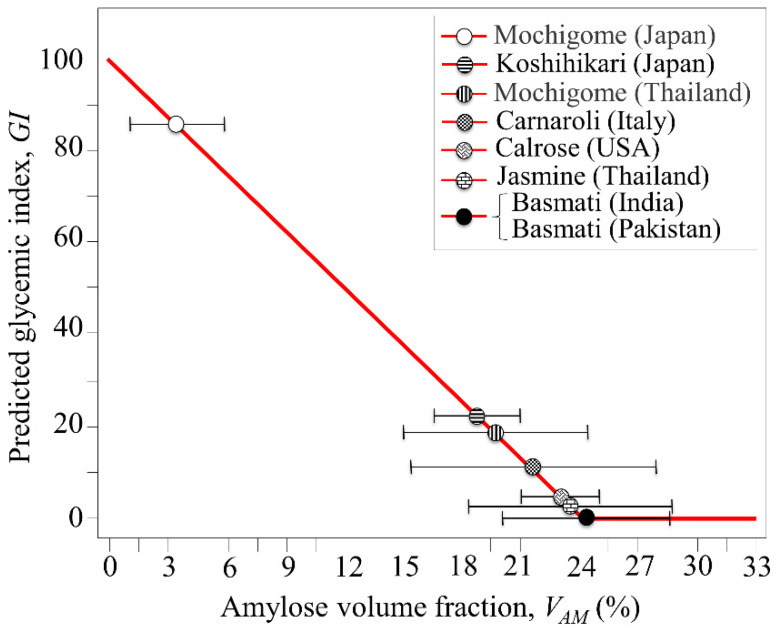
Predictive trend for postprandial glycemic index, *GI*, for eight rice cultivars (cf. labels) as a function of their amylose volume fraction, *V_AM_*, from Raman spectroscopy. The plot is based on clinical data from ref. [33].

In a previous study [7], we used a calibration plot provided by Jeevetha et al. [34] which gives the relationship between amylose content of white rice and glycemic index, GI, in order to estimate the glycemic impact of a series of popular Japanese cultivars. The study by Jeevetha et al. [34] was in agreement with previous reports by Cultivar-Miller et al. [35] and NikShanita et al. [36] which also showed an inverse relationship between amylose content and GI value, the higher the amylose content the lower the glycemic impact of rice. Based on these reference data and following our previous study on Japanese rice cultivars, we plotted a predictive trend of glycemic index, GI, for the eight rice cultivars investigated in this study as a function of their amylose volume fraction, *V_AM_*, as determined by Raman spectroscopy (cf. Section 3.2) (Figure 9). The plot in Figure 9 is quite instructive for the present purpose, but it can only be regarded as a semi-quantitative estimation of rice glycemic impact, given the well-known dependence of this parameter on human race [37] and the clinical conditions adopted to measure GI [38,39]. The plot in Figure 9 emphasizes the importance of polysaccharide composition in rice and its impact on human diet. The richest in amylopectin among non-glutinous cultivars was the Japanese Koshihikari cultivar, which accordingly was also the cultivar with the highest glycemic impact, followed by the Italian Carnaroli and the U.S. Calrose cultivars. The measured *V_AM_* and the predicted GI values of the Koshihikari cultivar from Kyoto Prefecture (i.e., the one studied here) were very close to those reported in [7] for other Koshihikari cultivars from Nagano, Hiroshima, and Fukushima Prefectures. On the other hand, the GI impact related to all long-grain cultivars was always very low (if any), due to their relatively high content of amylose. Accordingly, long-grain rice was generally confirmed as the most appropriate choice for controlling postprandial blood glucose levels. Despite its glutinous nature, the glutinous long-grain Thai mochigome was no exception to this trend, for its predicted GI being even lower than that of non-glutinous Japanese Koshihikari cultivar. On the other hand, the predicted glycemic impact of glutinous Japanese Mochigome was very close to that of pure glucose, due to its very low content of amylose.

### 4.2. Aromatic Amino Acids Contents and Protein Content/Structure

The aromatic amino acids phenylalanine and tryptophan are important building blocks for rice proteins and important precursors of secondary metabolism in the human body. As their supply only depends on nutritional provision, biosynthetic pathways have been searched for in order to accumulate them in rice at high concentrations [40,41]. Based on these accurate studies, new transgenic and mutant lines have been generated that are capable to accumulate phenylalanine and tryptophan in high concentrations through both genetic and metabolic engineering. The Koshihikari cultivar was found here to be quite rich in both aromatic amino acids with statistical relevance with respect to long-grain rice cultivars (cf. Figure 6). Besides genomic aspects and similar to the case of amylose contents, this characteristic could be affected by a number of production conditions (including climate, mineral, nutrition, etc.). Both content and composition of essential amino acids determine protein quality and favors more complete protein digestibility [42].

The sensory taste of rice is affected by several factors, which link to fundamental properties of its endosperm such as viscoelasticity and hardness [43]. From a structural chemistry viewpoint, the above characteristics are mainly related to both amylose and protein contents [44,45,46]. Lower amylose contents involve a more porous texture, which in turn renders stickier and softer the rice kernels, giving a better taste [47]. Together with starch factors, the abundance of phenylalanine and tryptophan free amino acids also represents an important compositional characteristic related to the taste of rice. The abundance of these two aromatic amino acids is a consequence of a specific metabolic pathway, which is referred to as glutamic-oxalacetic transaminease [48,49]. The contents of tryptophan and phenylalanine were found positively correlated with sensory taste in general, also confirming that larger concentrations of amino acid donors can further promote protein synthesis [48]. The above structural characteristics well explain why the Koshihikari cultivars have long dominated the Japanese (non-glutinous) rice ranking according to the Japan Grain Inspection Association [50]. Lower in amylose and higher in aromatic amino acid contents with respect to all long-grain non-glutinous rice cultivars, the Italian Carnaroli and the Calrose USA cultivars appear to near the Koshihikari one in terms of superior taste characteristics (cf. Figure 4 and Figure 6).

In line with studies previously published by these and other authors [8,51,52], the integrated intensity of the Amide I sub-bands can be used to give a reasonably accurate estimate of both protein fraction and fractions of protein secondary structures. Our quantitative Raman assessment of protein content (cf. Figure 8) indicated the Japanese Koshihikari cultivar as the richest one in protein content among non-glutinous rice cultivars, although no statistical significance could be found with respect to other non-glutinous cultivars. The total fraction of protein measured by Raman spectroscopy is in line with previously published studies. In the Koshihikari cultivar, the total amount of endosperm protein measured by Raman spectroscopy was ~5.9 wt.% (cf. Figure 8), which is in good agreement with the value 5.5 wt.% reported in the literature for the same cultivar according to nitrogen chemical analyses of milled kernels [53]. It is well known that the content of protein in rice grains is higher in the aleuronic layer than in the endosperm, as also found in our previous Raman assessments of rice kernel cross sections showing the presence of steep gradients in protein content as a function of distance from the kernel surface [8]. The present choice of a relatively large Raman probe (20× optical lens) was made in order to obtain average values over a relatively large area with a single measurement (thus minimizing the measurement time). However, this choice concurrently involved a penetration depth within individual kernels in the order of the tens of micron. The fractional values measured by the present Raman procedure are thus comprehensive of internal compositional gradients and, therefore, probe-dependent. Accordingly, in Raman measurements of rice, any comparison among different cultivars should be made by rigorously using the same incoming laser wavelength and laser probe configuration.

The Japanese Koshihikari cultivar contained the highest fraction of protein β-sheet (sub-band at 1638 cm^−1^) and random coil/β-turn secondary structures (sub-bands at 1674 and 1693 cm^−1^) with respect to the α-helix one (sub-band at 1658 cm^−1^). Fractions of specific protein secondary structures are known to relate to specific rice proteins. According to circular dichroism characterizations of purified rice proteins, Mawal et al. [54] reported that rice albumin is predominantly comprehensive of β-sheet and β-turn (Type II) structures, while no other rice protein possesses such a peculiar structure. Conversely, the secondary structure of rice prolamin is predominantly α-helical, while glutelin mainly exhibits the random coil structure accompanied by only a very minor fraction of α-helix. Note that random coil and α-helix relate to high and low protein digestibility, respectively. While the overall content of grain proteins determines rice nutritional quality, it is indeed the fractional amount of their secondary structures that gives a fingerprint for protein digestibility. While glutelin is generally the major protein contained in rice kernels (~80%) and prolamin a minor one (5–20%) [55], rice cultivars low in glutelin and high in prolamin are known to possess low protein digestibility and are preferred for patients with renal failure [56]. Prolamin is an indigestible component, which works as a resistant protein in the digestive tract. Rice cultivars high in prolamin (i.e., rich in α-helix) have also been shown to possess cholesterol-lowering properties [57]. The high fractions of random coil/β-turn and β-sheet structure in the Koshihikari cultivar detected by Raman spectroscopy are thus fingerprints for high protein digestibility (i.e., relative high in glutelin and low in prolamin) and high content of albumin, respectively. One could thus assume the random coil-to-α-helix Raman sub-band intensity ratio as a parameter for protein digestibility (i.e., Raman digestibility ratio, 0 ≤ *R_d_* ≤ 1), the higher *R_d_* the higher the protein digestibility. According to this criterion, the lowest protein digestibility among non-glutinous cultivars was found in Jasmine and Basmati (India) cultivars (*R_d_* = 0.40 and 0.44, respectively). Also the two investigated Japanese and Thai glutinous cultivars showed relatively low Raman protein digestibility ratios (i.e., *R_d_* = 0.50 and 0.59, respectively, vs. 0.91 of the Koshihikari cultivar, which was the cultivar with the highest protein digestibility among the studied ones). Both glutinous cultivars should thus be classified in the family of cultivars with low protein digestibility.

Abundance of albumin in rice kernels has been related to hypoglycemic and antioxidative properties. Albumin is a water-soluble protein with a hypoglycemic ability attributable to its α-amylase inhibiting activity [58,59]. The water-soluble amount of albumin represents ~15% of the total protein content in rice endosperm [60]. Rice albumin has been found to suppress the elevation of blood glucose and plasma insulin levels after oral glucose loading [61]. Regarding the antioxidant properties, rice albumin was found to provide a potent antioxidant action against low-density lipoprotein oxidation and maintained this property unchanged after digestion with trypsin and chymotrypsin [62]. In conclusion, the relatively high level of albumin of the Koshihikari cultivar could partly compensate for its relatively high glycemic impact due to an elevate content of amylopectin (cf. Figure 4), and also provides a superior antioxidant effect.

### 4.3. Comparison of Nutritional Facts Retrieved from Raman Spectroscopy

**Figure 10:**
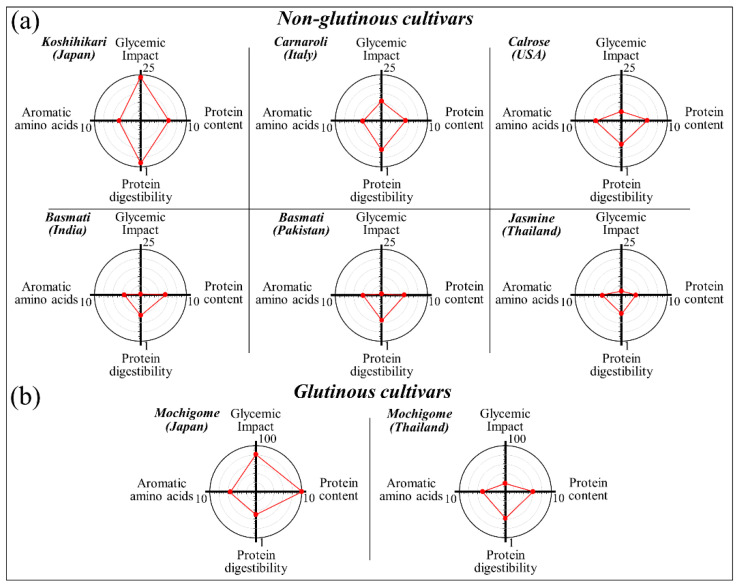
Polar diagrams comparing nutritional characteristics of non-glutinous (**a**) and glutinous (**b**) rice cultivars (cf. labels in inset); rotating clockwise from top: 0 ≤ *GI* ≤ 100 (glycemic impact, corresponding to the glycemic index (*GI*) relative to pure glucose), cumulative aromatic amino acid fraction, *W_Ph_* + *W_Tr_* (wt.%) (*W_Ph_* is the weight fraction of Phenylalanine and *W_Tr_* is the weight fraction of Tryptophan), protein fraction, *W_Pr_* (wt.%) as defined by Equation (6), and protein digestibility ratio 0 ≤ *R_d_* ≤ 1 (*R_d_* is the random coil-to-α-helix Raman sub-band intensity ratio).

According to the data presented in Section 3 and the notions described in the previous section, one could quantitatively assess and interpret specific features of the Raman spectrum of rice cultivars in terms of their nutritional traits. Four main parameters could be retrieved from the Raman spectrum, as follows: (i) the amylose content, which was converted into GI with respect to pure glucose (with score 100) and according to a calibration plot based on clinical assessments; (ii) cumulative weight fraction of aromatic phenylalanine and tryptophan amino acids (given in %) as a measure of protein quality and superior taste; (iii) weight fraction of proteins (given in %) as the nutritional counterpart of the carbohydrate content; and, (iv) random coil-to-α-helix fraction as a measure of protein digestibility (protein digestibility ratio, 0 ≤ *R_d_* ≤ 1). Polar diagrams comparing the above four parameters among the investigated non-glutinous and glutinous rice cultivars are shown in Figure 10a,b, respectively. The comparison reveals that the trend of the Japanese Koshihikari cultivar is unique among non-glutinous cultivars (the highest glycemic impact, GI, and protein digestibility, *R_d_*). On the other hand, both Carnaroli and Calrose cultivars appear more “balanced” with respect to both GI and R_d_. The three long-grain cultivars investigated (i.e., the two Basmati and the Jasmine cultivars) appear substantially equivalent: very low GI values vs. comparatively high *R_d_*. However, both Basmati cultivars were richer in protein content as compared to the Jasmine one. A comparison between the two investigated glutinous cultivars reveals significant differences both in terms of GI and protein content (much higher in the Japanese mochigome as compared to the Thai one). However, the two glutinous cultivars appeared to perform in a very similar way in terms of contents of aromatic amino acids and protein digestibility.

Besides summarizing the discussions given in the previous section, the polar diagrams in Figure 10 also show the power of Raman spectroscopy. This spectroscopic technique is capable to provide with a single measurement a wide spectrum of fundamental nutritional information through a non-contact and non-destructive procedure lasting only few tens of second. This study of internationally renowned rice cultivars, which confirms and extends our previous studies of Japanese cultivars [7,8], qualifying Raman spectroscopy as a precise and multitask biochemical method in food chemistry.

## 5. Conclusions

By using Raman spectroscopy for assessing chemical and structural components of rice, we compared in a fully non-destructive way Koshihikari and other internationally renowned rice cultivars of short and long-grain glutinous and non-glutinous rice. In the presented Raman characterizations, we applied and refined previously validated algorithms that can provide quantitative fractions of amylose, aromatic amino acids (phenylalanine and tryptophan), and proteins. Through these evaluations and with further morphological analyses of the Raman spectrum, we were able to quantify and discuss important nutritional characteristics, such as postprandial glycemic impact and protein digestibility. The nutritional parameters obtained by measuring Koshihikari and other international rice cultivars of both glutinous and non-glutinous types showed that the Japanese cultivar was relatively richer in protein contents, but also significantly higher in glycemic impact as compared to the other investigated cultivars, especially long-grain cultivars from Asian countries. An additional characteristic of non-glutinous Japanese Koshihikari cultivar was its high protein digestibility. More “balanced” in their nutritional properties were the Italian Carnaroli and the U.S. Calrose cultivars, which were significantly lower in *GI* as compared to the Japanese Koshihikari one, but yet relatively rich in proteins. Besides giving fundamental information for dietitians and nutritionists, the Raman spectrum of rice also contained important hints related to the taste of rice. Development of quantitative Raman calibrations linking chemical and structural characteristics of rice cultivars to their taste, stickiness, and hardness after cooking could be an interesting future task for researchers in the field of Raman spectroscopy applied to food chemistry.

## Data Availability

Data will be available on reasonable request to the authors.
